# Chest imaging features of patients afflicted with Influenza A (H1N1) in a Malaysian tertiary referral centre

**DOI:** 10.2349/biij.6.4.e35

**Published:** 2010-10-01

**Authors:** SI Bux, N Mohd. Ramli, S Ahmad Sarji, A Kamarulzaman

**Affiliations:** Department of Biomedical Imaging, Faculty of Medicine, University of Malaya, Kuala Lumpur, Malaysia

**Keywords:** Ground glass, swine flu, CXR, HRCT, A(H1N1)

## Abstract

This is a retrospective descriptive study of the chest imaging findings of 118 patients with confirmed A(H1N1) in a tertiary referral centre. About 42% of the patients had positive initial chest radiographic (CXR) findings. The common findings were bi-basal air-space opacities and perihilar reticular and alveolar infiltrates. In select cases, high-resolution computed tomography (CT) imaging showed ground-glass change with some widespread reticular changes and atelectasis.

## INTRODUCTION

In April 2009, a new global outbreak of a novel Influenza A virus, a swine virus with confirmed human infection, occurred. What is new about this virus is that it is unrelated to any swine influenza virus previously identified in North America. There is sustained human-to-human transmission in this new subtype of Influenza A virus [1]. It was termed Influenza A(H1N1) and 2009 H1N1 influenza by the United States Centre of Disease Control and Prevention (CDC) [2] and Pandemic H1N1/09 by the World Health Organization (WHO) [3]. The respiratory system is predominantly affected. Initial reported chest findings of A(H1N1) infections were bilateral diffuse lung opacities on chest radiographs (CXR). On computed tomography (CT), there were patchy ground glass opacities in both lungs with axial predominance and in the four lung quadrants consistent with acute respiratory distress syndrome [4]. To date, there has been no published data on CXR findings of patients with A(H1N1) in Malaysia. The authors report the CXR findings of 118 patients with confirmed A(H1N1).

## METHODOLOGY

Waiver of informed consent was obtained from the Hospital Ethics Committee due to public health care concern for information of this disease. A cross-sectional retrospective review of CXR and CT of the thorax of 166 patients with influenza symptoms who tested positive for Influenza A(H1N1) at the University of Malaya Medical Centre was done from end July 2009 to early September 2009. The majority of these patients were walk-in patients to the Accident and Emergency Unit who presented with constitutional influenza-like symptoms of fever, myalgia and headache. Laboratory confirmation of pandemic influenza A(H1N1) was carried out using a real-time polymerase chain reaction (PCR) protocol and primers from the CDC [5], and virus isolation in Madin-Darby canine kidney cells.

The decision to perform chest imaging in these patients was entirely at the discretion of the attending doctors at the Accident and Emergency Unit and, subsequently, doctors from the Respiratory and Infectious Disease Units based on the clinical severity of the patients. Out of the total, 48 patients including pregnant women were deemed not necessary to have any form of imaging. The chest radiographic images obtained from the 118 patients were reviewed by two staff radiologists, who have more than 10 years postgraduate experience, via consensus reading and the patterns recorded. Serial radiographs of patients with progressive clinical signs were also reviewed to assess disease progression and response to treatment by the Infectious Disease Unit team of doctors. Chest CT, which was performed on select cases based on severity and treatment response, were also read and the findings noted. The clinical data of the afflicted patients were obtained from the Hospital Infectious Disease Unit and compiled using Microsoft Office Excel (2003).

## FINDINGS

Of the patients presenting with influenza-like symptoms, 73 males and 93 females tested positive for Influenza A(H1N1) from end July to early September 2009 at the centre. Of the 118 patients who had chest radiographs, 56 were males and 62 were females. More than half of the patients (69/118) had normal chest radiographs. One of the patients who had a normal chest radiograph twice but remained symptomatic subsequently underwent a high-resolution CT (HRCT) examination a day after the second chest radiograph and the HRCT showed bi-apical fibrosis and ground-glass changes in the left lower lobe.

Among the abnormal chest radiographs (49/118), bilateral involvement (28/49) was more commonly noted. The commonest presentations were bi-basal air space infiltrates (12/49), perihilar mixed reticular and alveolar infiltrates (13/49) and unilateral basal air space opacity (6/49). Less common findings were round opacity (1/49), scattered focal air space opacity (4/49), linear opacities (2/49), linear opacities that progress to alveolar opacities (3/49), plethoric lungs (2/49), bronchiolitis (2/49) and pleural effusion (4/49). In the majority of patients (43/49), there was radiological improvement in the follow-up chest radiographs. Five had progressive alveolar consolidation ranging from 2 to 13 days with one developing acute respiratory distress syndrome (ARDS) according to the American-European Consensus Conference (AECC) criteria. Five patients had CT done 2 days to 1 week after the last chest radiograph because they showed poor clinical response to initial treatment. Three were HRCT and the common findings were widespread interstitial shadowing, ground-glass change and atelectasis. These were not detected on plain chest radiographs.

**Figure 1:**
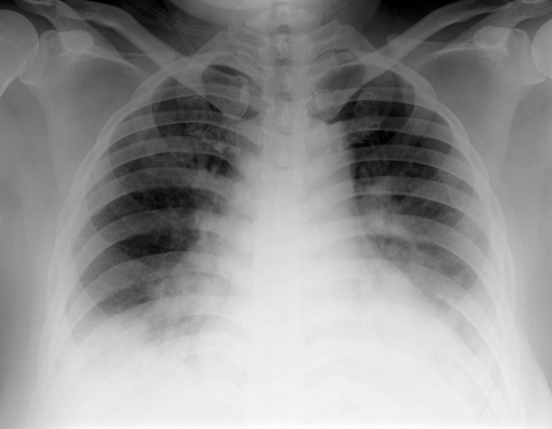
35-year-old Malay female with typical bi-basal air-space opacities.

**Figure 2:**
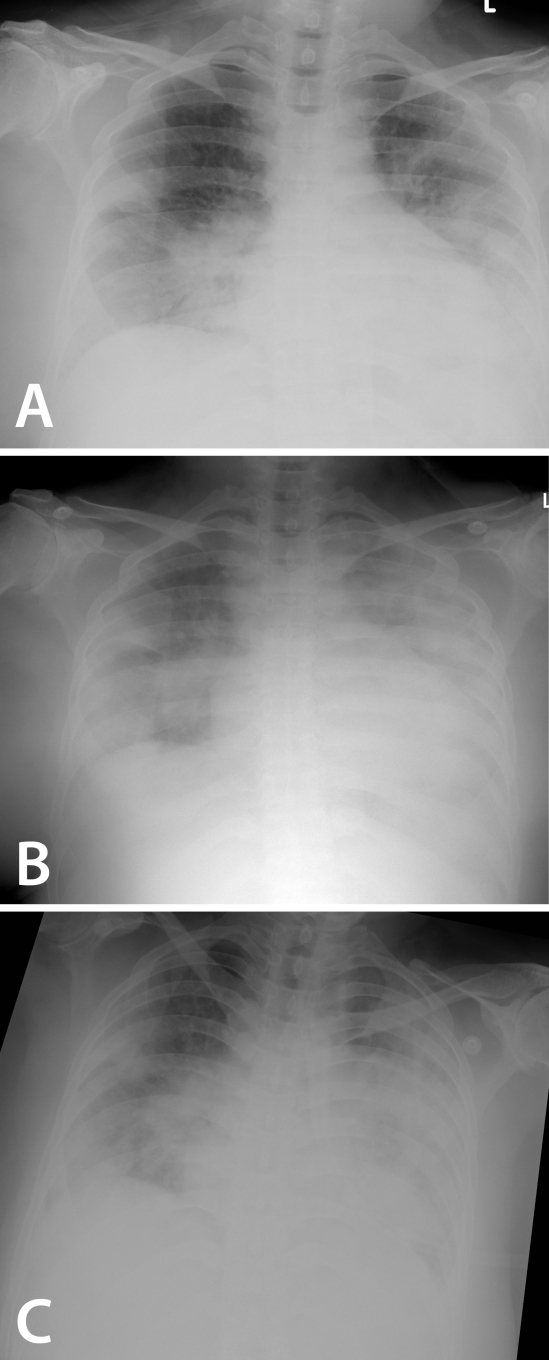
37-year-old female with progressive alveolar consolidation from (a) August 18, 2009; (b) August 21, 2009 and (c) August 24, 2009.

**Figure 3:**
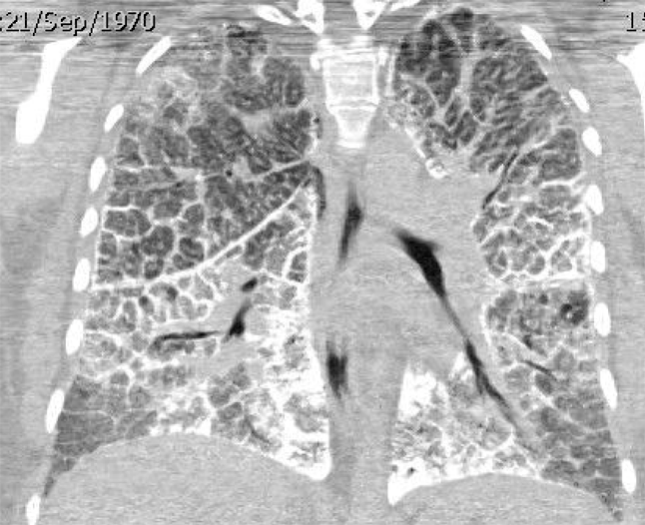
39-year-old female with reformatted coronal HRCT with diffuse interstitial and ground glass shadowing. No pleural effusion.

## DISCUSSION

H1N1 influenza took the world by storm. It emerged in Mexico during the influenza season of 2008-2009. Up to October 2, 2009, there has been 343,298 laboratory-confirmed cases of H1N1 influenza reported by the WHO with 4,108 deaths. This is an increase of 24,373 cases and 191 deaths since September 20, 2009 [6]. In Malaysia, the first confirmed case was a 21-year-old student who returned from Newark, USA, on a Malaysia Airlines flight MH091 on May 15, 2009. He presented with typical influenza symptoms – fever, sore throat and body aches. The first death occurred on July 24, 2009, where a 30-year-old Indonesian student who returned to Malaysia from a holiday break in Medan on July 5 had progressive fever and cough and eventually died [8]. As of October 17, 2009, there had been 77 H1N1-related deaths in Malaysia [7]. This made Malaysia the 36th country worldwide to be affected by the virus.

This short-term review highlights the chest radiographic findings in 118 patients with positive Influenza A(H1N1) from the centre. The bi-basal air space opacities seen correlate well with the findings of Influenza A(H1N1) in Mexico [4, 9]. Similar CXR findings are also seen in avian influenza such as sudden acute respiratory syndrome (SARS) and H5N1 [10, 11].

Currently, radiographic criterion is not included in the WHO diagnosis of Influenza A(H1N1). In this series, only 42% of patients demonstrate positive findings and this is likely due to the range of clinical presentation of the study population. Several studies have a similar initial CXR positive findings of 42-50% for this disease [12, 13].

In previous avian influenza outbreaks, such as SARS and H5N1 [10, 11, 14], CXR has been shown to be useful in the prognostication of the disease. According to the authors’ clinical and radiological observation of the Influenza A(H1N1), serial CXR revealed that lung infiltration increases with progression of the disease. This is similar to the findings of Ajlan et al where alveolar infiltration was predominant in cases that progressed to ARDS and showed near complete to total resolution in clinically resolving cases [14].

In CT, the most common reported change in Influenza A(H1N1) was ground glass shadowing. Other changes recorded on CT were focal or multifocal consolidation, interstitial changes and pulmonary embolism [4, 13, 14]. CT was valuable in detecting positive findings in the presence of normal CXRs and showed more extensive pattern of involvement when compared to CXR [14]. These findings were similar to the authors’ series although they did not find any evidence of pulmonary embolism in their sample population.

## CONCLUSION

The Influenza A (H1N1) CXR findings are similar to those reported for SARS and H5N1. Although imaging will play an important role in detecting lung changes, and to monitor disease progression as well as response to treatment; knowledge of the current epidemic status is essential for correct diagnosis.
